# Understanding the Relationship Between Severe Donor Diabetes Insipidus, Kidney Donor Profile Index, and Recipient Kidney Function

**DOI:** 10.1097/TXD.0000000000001916

**Published:** 2026-02-17

**Authors:** Lucia Calthorpe, Miguel Nunez, Gerardo Gamino, Timothy P. Copeland, Elaine Ku, Garrett R. Roll

**Affiliations:** 1 Department of Surgery, University of California, San Francisco, San Francisco, CA.; 2 School of Medicine, University of California, San Francisco, San Francisco, CA.; 3 Department of Epidemiology and Biostatistics, University of California, San Francisco, San Francisco, CA.; 4 Department of Medicine, University of California, San Francisco, San Francisco, CA.

## Abstract

**Background.:**

Severe diabetes insipidus (DI) causes dynamic changes in creatinine before organ donation. It is unknown whether terminal or peak creatinine is the optimal measure of graft quality among donors with severe DI.

**Methods.:**

Adult donors after brain death were identified from the United Network for Organ Sharing database, years 2015–2022. Kidney Donor Profile Index (KDPI) was computed using both terminal creatinine (KDPI-Terminal) and peak creatinine (KDPI-Peak). Model fit for the prediction of graft failure and reduced glomerular filtration rate (GFR) was compared. Logistic regression was used to determine the association between KDPI group reclassification and graft function.

**Results.:**

Of 28 718 kidney transplants, 29% of donors met criteria for severe DI. No differences in estimated GFR (eGFR) or all-cause graft failure were observed on the basis of the presence of DI. Among donors with severe DI, KDPI-Peak improved prediction of reduced eGFR at 1 y (eGFR <60 mL/min/1.73 m^2^, eGFR <30 mL/min/1.73 m^2^). Additionally, patients who received organs from donors with severe DI whose KDPI group would be reclassified on the basis of the use of peak rather than terminal creatinine had 2-fold increased odds of reduced eGFR at 1 y (eGFR <30 mL/min/1.73 m^2^; odds ratio, 1.92; 95% confidence interval, 1.37-2.69).

**Conclusions.:**

This national analysis confirms that while the presence of severe DI alone is not associated with graft outcomes, the use of peak creatinine significantly improves risk stratification of donor graft quality. Precise risk stratification is critical for efforts to improve appropriate kidney allocation and increase confidence in organ function after transplant.

## Graphical abstract

**Figure:**
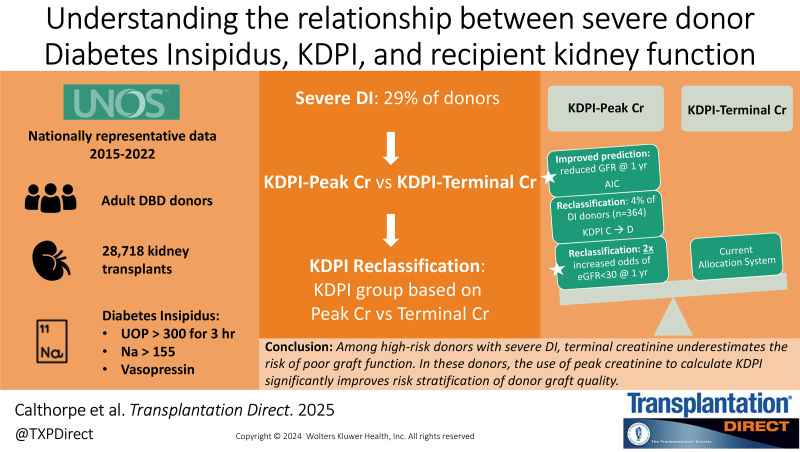

## INTRODUCTION

Severe diabetes insipidus (DI) is common before organ procurement, occurring in 49%–60% of donors with brain death.^1^ The hypothalamic-pituitary failure that precedes DI in patients with brain death triggers electrolyte shifts, dehydration, and hypovolemia. Management of DI in donors involves fluid resuscitation and vasopressin administration to prevent hypotension and kidney injury.^2,3^ However, in the days leading up to procurement, supraphysiologic urine output and these corrective measures contribute to dynamic changes in creatinine.^4,5^

Rates of chronic disease in the donor pool continue to rise, but there have been no recent advancements in kidney quality assessment.^6^ Nonutilization of kidneys from deceased donors is an ongoing problem driven primarily by a lack of confidence in our ability to predict which kidneys will have poor function after transplant. Terminal creatinine is widely used as a measure of kidney function in donors and is included in the Kidney Donor Profile Index (KPDI), a percentile score incorporating 10 donor characteristics to assess graft quality and project long-term outcomes.^7^ It is unclear whether terminal creatinine is the best available predictor of graft quality among donors with severe DI. Previously, we compared the use of peak creatinine to terminal creatinine to calculate KDPI in kidneys from donors with severe DI. We found that the use of peak creatinine improved the prediction of posttransplant estimated glomerular filtration rate (eGFR).^8^ Notably, donors with severe DI who would be reclassified into a higher KDPI category based on peak rather than terminal creatinine had 2.5 times greater odds of reduced GFR 1 y posttransplant. That study was limited by its single-center design, but it did suggest a pathway to increasing confidence in the assessment of deceased donor kidneys, thereby reducing organ nonutilization.^8^

The present study uses national data to examine the question: how do changes in serum creatinine in the setting of severe DI affect the accuracy of the KDPI in predicting graft function? Specifically, we aim to (1) compare the use of peak (KDPI-Peak) and terminal (KDPI-Terminal) creatinine to predict the risk of graft failure and reduced eGFR at 1 y posttransplant; and (2) understand the association between KDPI group reclassification (with the use of peak rather than terminal creatinine) and outcomes, including reduced 1-y eGFR and graft failure.

## MATERIALS AND METHODS

### Data Source

National data were extracted from the United Network for Organ Sharing database.^9^ Analysis was restricted to adult patients who received kidneys from patients who donated after brain death between 2015 and 2022.^9^ Patients who received a kidney from a donation after circulatory death were excluded because of the variable degree of acute kidney injury resulting from the donation process, which could confound results.

KDPI is divided into 4 groups for organ allocation: KDPI-A: ≤20%; KDPI-B: 21%–35%; KDPI-C: 36%–84%; and KDPI-D: 85%–100%.^10^ Analysis was restricted to patients who received kidneys from donors with KDPI >35%. We chose to focus this analysis on higher-risk donors, as fluctuations in creatinine among lower KDPI grafts are unlikely to signify chronic kidney disease, and therefore less frequently affect clinical decision-making and transplant outcomes. In contrast, in higher-risk kidneys, it is important to attempt to achieve an accurate assessment of graft quality so the organ can be allocated to an appropriate recipient with confidence. Therefore, the threshold of >35% was chosen for KDPI as it represents groups C and D.^10^

### Diabetes Insipidus

Consistent with the existing literature in nondonor populations, DI was definedon the basis of urine output, serum sodium, and vasopressin administration.^11^ Specifically, donors were classified as having severe DI if they had hourly urine output >300 mL/h for ≥3 h, concurrent with serum sodium >155 mmol/L and vasopressin administration in the final 24 h before donation.^11,12^ The terms “severe DI” and “no/limited DI” are used to differentiate between donors who do and do not meet this criterion. This distinction recognizes that many donors not meeting the threshold still exhibit some degree of DI physiology, and there are probably few patients with brain death who do not have any DI. In addition, a sensitivity analysis was performed, considering an alternative definition of severe DI with a higher urine output threshold (500 mL/h for ≥3 h, concurrent with serum sodium >155 mmol/L and vasopressin administration in the final 24 h before donation).^11^

### Covariates

Donor characteristics included age, sex, body mass index (BMI), history of hypertension, peak sodium, peak and terminal creatinine, cause of death (anoxia, cerebrovascular accident/stroke, head trauma, central nervous system tumor), and KDPI (2022 scaling factor).^7,13^ Covariates included recipient age, sex, BMI, history of diabetes, and panel-reactive antibody. Analyses were additionally adjusted for cold ischemia time.

### Outcomes

The primary outcome of this analysis was reduced eGFR after transplant, defined as eGFR <60 mL/min/1.73 m^2^, and eGFR <30 mL/min/1.73 m^2^ at 1 y posttransplant using the Chronic Kidney Disease Epidemiology Collaboration Creatinine Equation (2021).^14,15^ eGFR was additionally analyzed as a continuous outcome. Time to graft failure (censored for death with a functioning graft) was considered as a secondary outcome.

### Statistical Analysis

Organ donors were classified by the presence of severe DI, and continuous variables were summarized using medians with interquartile ranges. Categorical variables were expressed as percentages. KDPI was calculated for each donor kidney, using 2022 as the reference population.^13^ KDPI was categorized as follows: C: 36%–84%, D: 85%–100%, consistent with groups used for organ allocation.^10^ Next, an alternative KDPI was calculated on the basis of peak rather than terminal creatinine, designated KDPI-Peak. The Akaike Information Criterion (AIC) was used to compare model fit between multivariable logistic and linear regression models using KDPI-Peak versus KDPI-Terminal to predict reduced eGFR, stratifying by the presence of severe DI.^16^ Next, we repeated our analyses using multivariable Cox proportional hazards models to compare the prediction of graft failure between KDPI-Peak and KDPI-Terminal. AIC was used to compare the risk discrimination between the peak and terminal KDPI scores.

Patients whose KDPI group would be reclassified (ie, KDPI-C to KDPI-D) on the basis of the use of peak creatinine (versus terminal creatinine) were identified. Multivariable logistic and linear regression models were used to investigate the associations between KDPI reclassification and impaired graft function at 1 y posttransplant. Similarly, multivariable Cox proportional hazards models were used to investigate whether reclassification based on peak creatinine among patients with severe DI was associated with an increased risk of graft failure.

The following sensitivity analyses were performed. First, the reclassification analysis was performed on a broader cohort of all adult patients who received a kidney donation after brain death, irrespective of donor KDPI (ie, groups A–D versus C–D). An interaction analysis was performed to test our hypothesis that the effect of reclassification would be most pronounced among patients who received kidneys from donors with higher baseline KDPI scores. A second sensitivity analysis was performed, considering a more conservative definition of severe DI with a higher urine output threshold.^11^ Finally, a sensitivity analysis was performed on the outcome of eGFR at 2 y posttransplant.

A complete case analysis was performed. STATA/IC version 16.1 was used for analyses, and statistical significance was set at a *P* value of <0.05.^17^ Given that the study was based on an existing de-identified database, this study was considered exempt by the University of California, San Francisco Institutional Review Board, and the need for written informed consent was waived (IRB No. 20-31396). This study was conducted in accordance with the Strengthening the Reporting of Observational Studies in Epidemiology guidelines for observational studies.^18^

## RESULTS

### Recipient and Donor Characteristics

Table 1 summarizes the clinical and demographic characteristics of the cohort, stratified by the presence of severe DI. Overall, 29% of the transplanted kidneys came from donors with severe DI. The median age was 60 among recipients of kidneys from the limited/no DI group, and it was 59 from the severe DI group (*P* < 0.001). Estimates for recipient sex, BMI, diabetes, median panel-reactive antibody, and cold ischemia time were similarly distributed across groups.

**TABLE 1.** Characteristics of the 28 718 kidney transplant recipients and kidney transplant organs

	Limited DI	Severe DI	*P*
	N = 20 481 (71%)	N = 8237 (29%)	
Recipient characteristics			
Age, y	60 (51–66)	59 (51–66)	**<0.001**
Female	40	41	0.41
BMI, kg/m^2^	28 (24–32)	28 (25–32)	**<0.001**
Diabetes	45	47	**0.03**
PRA	0 (0–22)	0 (0–31)	**<0.001**
CIT, h	18 (13–24)	18 (12–23)	**<0.001**
Donor organ characteristics			
Age, y	50 (41–57)	50 (42–56)	0.62
Female	45	48	**<0.001**
BMI, kg/m^2^	28 (23–33)	28 (24–33)	**0.01**
HTN	49	50	**0.03**
Peak sodium, mmol/L	154 (150–61)	162 (158–168)	**<0.001**
Peak Cr, mg/dL	1.5 (1.1–2.3)	1.4 (1.1–1.9)	**<0.001**
Delta Cr (peak Cr–terminal Cr)	0.23 (.06–0.5)	0.28 (0.1–0.5)	**<0.001**
Cause of death			**<0.001**
Other	2	2	
Anoxia	43	30	
CVA/stroke	37	48	
Head trauma	17	20	
CNS tumor	<1	<1	
KDPI group			**<0.001**
C	88	90	
D	12	10	
Outcomes			
eGFR <60 mL/min/1.73 m^2^, 1 y	63	62	0.19
eGFR <30, 1 y	9	8	0.12
eGFR, 1 y	53.1 (40.7–67.9)	53.7 (41.5–67.8)	0.18
Graft failure	4	4	0.14

Data presented as median (interquartile range) or percent.

Bold values indicate statistical significance.

DI, diabetes insipidus; BMI, body mass index; CIT, cold ischemia time; CNS, central nervous system; eGFR, estimated glomerular filtration rate; Cr, creatinine; CVA, cerebrovascular accident; HTN, hypertension; KDPI, Kidney Donor Profile Index; PRA, panel-reactive antibody.

In terms of donor characteristics, median age was equivalent across groups (50 y), 48% of donors with severe DI were women, compared with 45% of donors with limited DI (*P* < 0.001). Peak sodium was higher in the severe DI group (median 162 versus 154, *P* < 0.001). Although peak creatinine was observed to be higher in the limited DI group, the change in creatinine between peak and terminal was larger in patients with severe DI (0.28 versus 0.23, *P* < 0.001). Statistically significant differences were observed by cause of death, with a higher prevalence of cerebrovascular accident/stroke in the severe DI group (*P* < 0.001). The incidence of graft failure and the prevalence of reduced graft function at 1 y were not significantly different across groups (eg, graft failure: 4.4% among limited/no DI versus 4.1% among severe DI, *P* = 0.14). Median eGFR at 1 y posttransplant was 53.1 mL/min/1.73 m^2^ among patients who received kidneys from donors with limited/no DI, compared with 53.7 mL/min/1.73 m^2^ for donors with severe DI (*P* = 0.18).

### Peak Versus Terminal Creatinine

Table 2 compares the associations between KDPI-Peak and KDPI-Terminal for the prediction of eGFR <60 mL/min/1.73 m^2^ and eGFR <30 mL/min/1.73 m^2^ at 1 y after transplant, stratified by the presence of severe DI. For recipients whose donors had both limited/no DI and severe DI, models with KDPI-Peak had lower AIC values compared with KDPI-Terminal (AIC for eGFR <30 mL/min/1.73 m^2^ among patients with severe DI: 4661 versus 4671), indicating superior model performance (Table 2). Similarly, KDPI-Peak demonstrated improved model fit relative to KDPI-Terminal for predicting eGFR at 1 y as a continuous outcome (AIC 72 040 versus 72 094, patients with severe DI). Furthermore, KDPI-Peak demonstrated evidence of improved model fit for predicting eGFR at 2 y posttransplant, relative to KDPI-Terminal (**Table S1, SDC,** https://links.lww.com/TXD/A834).

**TABLE 2.** Comparison of KDPI-Peak vs KDPI-Terminal for predicting reduced eGFR and graft failure (N = 28 718)

	Limited DI			Severe DI		
	OR, HR, coef.	95% CI, *P*	AIC	OR, HR, coef.	95% CI, *P*	AIC
eGFR <60 mL/min/1.73 m^2^, 1 y						
KDPI-Terminal			25 977			10 670
C	REF			REF		
D	2.21	(1.99-2.45) <0.001		2.17	(1.82-2.58) <0.001	
KDPI-Peak			25 943			10 634
C	REF			REF		
D	2.10	(1.92-2.30) <0.001		2.22	(1.92-2.58) <0.001	
eGFR <30 mL/min/1.73 m^2^, 1 y						
KDPI-Terminal			12 064			4671
C	REF			REF		
D	2.30	(2.03-2.60) <0.001		2.40	(1.94-2.96) <0.001	
KDPI-Peak			12 057			4661
C	REF			REF		
D	2.18	(1.94-2.45) <0.001		2.34	(1.93-2.84) <0.001	
eGFR continuous, 1 y						
KDPI-Terminal			180 887			72 094
C	REF			REF		
D	–7.82	(–8.69 to –6.95) <0.001		–8.60	(–10.01 to –7.20) <0.001	
KDPI-Peak			180 858			72 040
C	REF			REF		
D	–7.30	(–8.07 to –6.52) <0.001		–8.80	(–10.03 to –7.58) <0.001	
Graft failure						
KDPI-Terminal			16 476			5410
C	REF			REF		
D	1.91	(1.60-2.29) <0.001		1.86	(1.35-2.57) <0.001	
KDPI-Peak			16 478			5408
C	REF			REF		
D	1.77	(1.50-2.09) <0.001		1.81	(1.36-2.41) <0.001	

Adjusted for: recipient sex, age, body mass index, diabetes, panel-reactive antibody, and cold ischemia time.

Bold values indicate statistical significance.

AIC, Akaike Information Criterion; CI, confidence interval; DI, diabetes insipidus; eGFR, estimated glomerular filtration rate; HR, hazard ratio; KDPI, kidney donor profile index; OR, odds ratio.

When comparing the AIC between multivariable Cox proportional hazards models using KDPI-Peak versus KDPI-Terminal for the prediction of graft failure, similar AICs were observed for the KDPI-Peak and KDPI-Terminal models among recipients whose donor had severe DI (AIC 5408 versus 5410), and those with limited/no DI (AIC 16 478 versus 16 476; Table 2).

### Reclassification of KDPI

**Figure 1.**
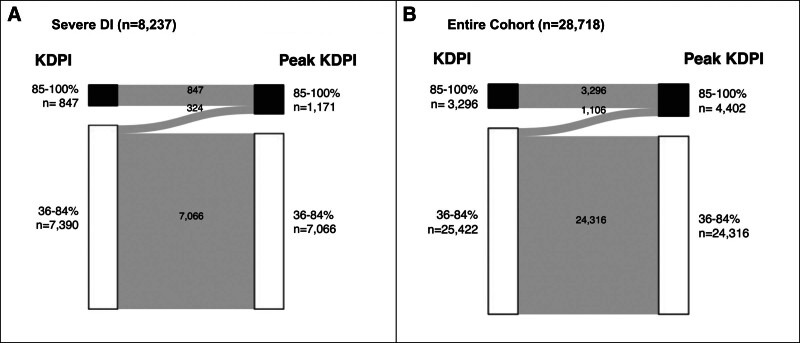
Change in KDPI based on peak vs terminal creatinine (n = 28 718). A, Change in KDPI from group C to D among donors with severe DI (n = 8237). B, Change in KDPI from group C to D among the entire cohort (n = 28 718). DI, diabetes insipidus; KDPI, Kidney Donor Profile Index.

Using KDPI-Peak instead of KDPI-Terminal in donors with severe DI would reclassify 324 kidneys to a higher allocation group, increasing the proportion of KDPI-D patients by 38% (Figure 1). In donors with severe DI, the odds of an eGFR <60 mL/min/1.73 m^2^ at 1 y was 2-fold higher when the donor would have been reclassified into a higher group using KDPI-Peak instead of KDPI-Terminal (odds ratio [OR], 2.15, 95% confidence interval [CI], 1.65-2.80; *P* < 0.001; Table 3). Similarly, the odds of eGFR <30 mL/min/1.73 m^2^ at 1 y were significantly increased among donors who would have been reclassified (OR, 1.92; 95% CI, 1.37-2.69; *P* < 0.001). When analyzed as a continuous outcome, reclassification was associated with a reduction in eGFR of 8.1 mL/min/1.73 m^2^ among donors with severe DI (*P* < 0.001). The hazard ratio for graft failure was 1.54 among donors who would have been reclassified; however, this effect did not reach statistical significance (*P* = 0.105). Results across the entire cohort were similar; however, point estimates for effect size were generally smaller (Table 3). When 2-y eGFR outcomes were analyzed, we found evidence of a similarly elevated risk of reduced eGFR with KDPI reclassification (OR, 2.43; 95% CI, 1.76-3.36; *P* < 0.001 for eGFR <60 mL/min/1.73 m^2^ at 2 y; **Table S2, SDC,** https://links.lww.com/TXD/A834).

**TABLE 3.** Effect of KDPI reclassification on graft function, KDPI >35%

	Entire cohort (N = 28 718)			Severe DI (N = 8237)		
	OR, HR, coef.	95% CI	*P*	OR, HR, coef.	95% CI	*P*
eGFR <60 mL/min/1.73 m^2^, 1 y	**1.81**	**(1.58 to 2.08**)	**<0.001**	**2.15**	**(1.65 to 2.80**)	**<0.001**
eGFR <30 mL/min/1.73 m^2^, 1 y	**1.71**	**(1.42 to 2.06**)	**<0.001**	**1.92**	**(1.37 to 2.69**)	**<0.001**
eGFR, mL/min/1.73 m^2^, 1 y	**–5.79**	**(–6.99 to –4.59**)	**<0.001**	**–8.10**	**(–10.24 to –5.95**)	**<0.001**
Graft failure	**1.37**	**(1.03 to 1.81**)	**0.028**	1.54	(0.91 to 2.61)	0.105

Models adjusted for: recipient sex, age, BMI, diabetes, PRA, cold ischemia time, and KDPI group.

CI, confidence interval; DI, diabetes insipidus; eGFR, estimated glomerular filtration rate; HR, hazard ratio; KDPI, Kidney Donor Profile Index; OR, odds ratio.

Sensitivity analysis was performed to assess the effect of reclassification across the full KDPI range (ie, analytical cohort with KDPI-A and -B donors added; **Table S3, SDC,** https://links.lww.com/TXD/A834). Among this cohort, using peak rather than terminal creatinine would have reclassified 3828 (20%) kidneys into a higher allocation group. We found similar evidence of increased risk of reduced eGFR at 1 y posttransplant among donors who would have been reclassified; however, with a smaller effect size (eg, eGFR <60 mL/min/1.73 m^2^ at 1 y OR, 1.41; 95% CI, 1.30-1.53; *P* < 0.001). Tests for interaction revealed a significantly larger effect size among donors with baseline KDPI >35%.

### Alternative Criteria for Severe DI

An additional sensitivity analysis was performed, considering an alternative criterion for severe DI (urine output 500 mL/h for ≥3 h, concurrent with serum sodium >155 mmol/L and vasopressin administration in the final 24 h before donation). Findings are summarized in **Tables S4 and S5** (**SDC,** https://links.lww.com/TXD/A834). Overall, we find evidence of improved model fit for KDPI-Peak compared with KDPI-Terminal for prediction of reduced GFR at 1 y among patients with severe DI (AIC: 4510 versus 4521, eGFR <60 mL/min/1.73 m^2^; AIC: 30 038 versus 30 052 for eGFR as a continuous outcome; **Table S4, SDC,** https://links.lww.com/TXD/A834). Furthermore, we find evidence of a significant association between KDPI group reclassification at reduced eGFR at 1 y (OR, 2.16; 95% CI, 1.35-3.46; *P* = 0.001 for eGFR <60 mL/min/1.73 m^2^ among donors with severe DI; **Table S5, SDC,** https://links.lww.com/TXD/A834).

## DISCUSSION

This analysis of national kidney transplant data suggests that among donors with severe DI, terminal creatinine underestimates the risk of impaired eGFR posttransplantation. Specifically, we found a 2-fold increase in the risk of impaired kidney function (eGFR <60 mL/min/1.73 m^2^, eGFR <30 mL/min/1.73 m^2^), among patients who received kidneys from donors with severe DI who would have been classified into a higher KDPI group with the use of peak creatinine. Further, we observed improved prediction of impaired graft function with the use of peak creatinine (versus terminal creatinine) among patients who received a kidney from a donor with severe DI. These findings highlight the notion that terminal creatinine incompletely captures donor quality in the setting of severe DI.

Although DI is highly prevalent among donors after brain death, the effect of severe DI on metrics used to guide graft allocation has largely not been investigated.^19,20^ The standard management of DI before donation involves the administration of vasopressin and fluid boluses to offset the massive amount of urine being produced.^2,4,20^ These interventions, in combination with fluid shifts, can cause significant fluctuations in creatinine leading up to donation. Specifically, it is likely that these interventions dilute serum creatinine, potentially overestimating donor kidney function. KDPI is a tool comprising 10 characteristics that provides an estimate of graft survival and is therefore used to allocate kidneys to the most appropriate pool of potential recipients.^7^ However, KDPI relies on terminal creatinine, which may be impacted by the aforementioned physiologic derangements. The key finding of this study is that there is a small but significant subset of donors with severe DI for whom terminal creatinine significantly overestimates graft function. Although the presence of severe DI alone does not predict poor graft function, it does complicate the assessment of organ function. This is important in the context of increasing rates of chronic disease in the pool of potential donors and a clear lack of confidence in our ability to predict kidney function after transplant based on the ongoing rate of kidney nonutilization.

Previously, our group conducted a single-center analysis comparing peak to terminal creatinine among donors with severe DI after brain death.^8^ We found that while the presence of severe DI was not associated with outcomes after transplant, patients whose KDPI group would have been reclassified on the basis of the use of peak creatinine had an elevated risk of reduced graft function.^8^ The present analysis expands on this work in several important ways. First, while our previous analysis was based on data drawn from a single center, the present analysis uses nationally representative data. Second, our previous analysis was limited because we did not have data on urine output to incorporate into the definition of DI. In the present analysis, we incorporated granular hourly urine output data into our criteria for DI.

We conducted a sensitivity analysis to examine the effect of reclassification across the full range of KDPI scores. We found evidence of a significant effect of reclassification across the board, albeit with a smaller effect size. Furthermore, we identified a statistically significant interaction with baseline KDPI, supporting the notion that reclassification is particularly important for predicting poor outcomes in our original cohort (KDPI >35%).

Prior studies have investigated the use of terminal creatinine to predict graft function following transplantation. For example, a recent analysis of United Network for Organ Sharing data compared the use of initial versus terminal creatinine to calculate KDPI among all deceased donor kidneys (both donation after brain death and donation after circulatory death).^21^ The authors found no significant differences in the prediction of transplant failure (assessed with area under the receiver operating characteristic curve) between initial and terminal creatinine.^21^ This result is consistent with other literature, which has demonstrated that acute kidney injury in donors is not associated with adverse transplant outcomes.^22–26^ It is important to note that this body of literature differs from the present analysis in several important ways. Specifically, our analysis focuses on a subpopulation of donors after brain death who develop severe DI. Overall, our results suggest that terminal creatinine is inadequate to predict poor function in donors with severe DI, given the physiologic changes.

There are several important limitations to this study. First, we acknowledge that DI is highly prevalent among donors with brain death, and characterizing the severity of DI is challenging based on currently captured data. This may explain our findings of generally lower AIC values for KDPI-Peak relative to KDPI-Terminal, even among “limited DI” donors, and the fact that the median peak sodium level among “limited DI” donors was as high as 154 mmol/L. Second, we acknowledge that KDPI is a relative measure. If all patients were to be categorized on the basis of peak rather than terminal creatinine, there would be an increase in KDPI values across the board, affecting percentile groups. It is also possible that an increase in KDPI scores with the use of peak rather than terminal creatinine could, if misinterpreted, increase the organ discard rate. However, we believe this risk is balanced by the benefit of more precise risk stratification, which is critical for efforts to improve appropriate kidney allocation and increase confidence in organ function after transplant. Further, the overall percentage of kidneys that would be reclassified between groups C and D is relatively small (4% among those with severe DI in our cohort). In addition, we recognize that serum creatinine is an imperfect measure of kidney function, and more granular data on donor characteristics or alternative measures of kidney function are not available in this national database. Despite the known limitations of creatine, our current allocation system still relies on it as a key component of KDPI. To reduce nonutilization, we need to increase confidence in organ assessment. Our findings highlight the presence of a small but significant proportion of donors with severe DI who have a large change in KDPI driven by DI, and for whom terminal creatinine substantially underestimates the risk of poor graft outcomes.

## CONCLUSIONS

Among high-risk donors with severe DI, terminal creatinine underestimates the risk of poor graft function, as evidenced by the 2-fold increase in reduced eGFR associated with KDPI reclassification based on peak creatinine. Although the presence of severe DI itself was not associated with graft outcomes, the impact of its associated physiologic derangements on creatinine levels before organ procurement should be considered in metrics used to guide organ allocation. Specifically, the use of peak rather than terminal creatinine for KDPI calculation should be considered for donors with severe DI to ensure the most appropriate matching of donors to waitlisted patients, increase confidence in organ function, and increase kidney transplantation rates.

## Supplementary Material



## References

[1] Nair-Collins M Northrup J Olcese J. Hypothalamic-pituitary function in brain death: a review. J Intensive Care Med. 2016;31:41–50. 24692211 10.1177/0885066614527410

[2] Meyfroidt G Gunst J Martin-Loeches I. Management of the brain-dead donor in the ICU: general and specific therapy to improve transplantable organ quality. Intensive Care Med. 2019;45:343–353. 30741327 10.1007/s00134-019-05551-y PMC7095373

[3] Opdam HI. Hormonal therapy in organ donors. Crit Care Clin. 2019;35:389–405. 30784617 10.1016/j.ccc.2018.11.013

[4] Tanim Anwar ASM Lee JM. Medical management of brain-dead organ donors. Acute Crit Care. 2019;34:14–29. 31723901 10.4266/acc.2019.00430 PMC6849043

[5] Franz S Skopp G Boettcher M. Creatinine excretion in consecutive urine samples after controlled ingestion of water. Drug Test Anal. 2019;11:435–440. 30276981 10.1002/dta.2514

[6] Hardgrave H Jeon H Wells A. Kidney utilization in the context of a shifting donor landscape in the United States. Transplantation. 2024;109:e311–e316. 40101109 10.1097/TP.0000000000005229

[7] Rao PS Schaubel DE Guidinger MK. A comprehensive risk quantification score for deceased donor kidneys: the kidney donor risk index. Transplantation. 2009;88:231–236. 19623019 10.1097/TP.0b013e3181ac620b

[8] Nunez M Gardner J Syed S. Diabetes insipidus in deceased donors and outcomes in kidney transplant recipients. Am J Kidney Dis. 2024;84:129–132. 10.1053/j.ajkd.2023.10.015 38160701

[9] Organ Procurement and Transplantation Network. National data. Available at https://optn.transplant.hrsa.gov/data/view-data-reports/national-data/. Accessed December 28, 2024.

[10] Organ Procurement and Transplantation Network. Kidney Donor Profile Index (KDPI) guide for clinicians. Available at https://optn.transplant.hrsa.gov/professionals/by-topic/guidance/kidney-donor-profile-index-kdpi-guide-for-clinicians/. Accessed December 28, 2024.

[11] de Vries F Lobatto DJ Verstegen MJT. Postoperative diabetes insipidus: how to define and grade this complication? Pituitary. 2021;24:284–291. 32990908 10.1007/s11102-020-01083-7 PMC7966184

[12] Hadjizacharia P Beale EO Inaba K. Acute diabetes insipidus in severe head injury: a prospective study. J Am Coll Surg. 2008;207:477–484. 18926448 10.1016/j.jamcollsurg.2008.04.017

[13] Organ Procurement and Transplantation Network. KDRI to KDPI Mapping Table. Available at https://optn.transplant.hrsa.gov/media/wnmnxxzu/kdpi_mapping_table.pdf. Accessed December 28, 2024.

[14] Miller WG Kaufman HW Levey AS. National Kidney Foundation Laboratory Engagement Working Group recommendations for implementing the CKD-EPI 2021 race-free equations for estimated glomerular filtration rate: practical guidance for clinical laboratories. Clin Chem. 2022;68:511–520. 34918062 10.1093/clinchem/hvab278

[15] National Kidney Foundation. eGFR calculator. Available at https://www.kidney.org/professionals/gfr_calculator. Accessed December 28, 2024.

[16] Sutherland C Hare D Johnson PJ. Practical advice on variable selection and reporting using Akaike Information Criterion. Proc Biol Sci. 2023;290:20231261. 37752836 10.1098/rspb.2023.1261 PMC10523071

[17] StataCorp. Stata statistical software: release 16. StataCorp LLC; 2019.

[18] Checklists - STROBE. Available at https://www.strobe-statement.org/checklists/. Accessed June 20, 2025.

[19] Nair-Collins M Northrup J Olcese J. Hypothalamic–pituitary function in brain death. J Intensive Care Med. 2014;31:41–50. 24692211 10.1177/0885066614527410

[20] Yaqoob M Weiß M Rücker F. Incidence and treatment of arginine vasopressin deficiency (Central Diabetes Insipidus) in the setting of brain death and associations with renal function and hemodynamics in organ donors. J Clin Med. 2024;13:7073. 39685532 10.3390/jcm13237073 PMC11642225

[21] Chiles MC Husain SA Skillen W. Predictive value of using initial versus terminal deceased donor creatinine to calculate the kidney donor risk index. Am J Kidney Dis. 2017;70:153–154. 28416320 10.1053/j.ajkd.2017.02.373

[22] Hall IE Akalin E Bromberg JS. Deceased-donor acute kidney injury is not associated with kidney allograft failure. Kidney Int. 2019;95:199–209. 30470437 10.1016/j.kint.2018.08.047 PMC6331055

[23] Liu C Hall IE Mansour S. Association of deceased donor acute kidney injury with recipient graft survival. JAMA Netw Open. 2020;3:e1918634. 31913491 10.1001/jamanetworkopen.2019.18634 PMC6991314

[24] Hall IE Akalin E Bromberg JS. Deceased-donor acute kidney injury is not associated with kidney allograft failure. Kidney Int. 2019;95:199–209. 30470437 10.1016/j.kint.2018.08.047 PMC6331055

[25] Yu CC Ho HC Yu TM. Kidneys from standard-criteria donors with different severities of terminal acute kidney injury. Transplant Proc. 2014;46:3335–3338. 25498047 10.1016/j.transproceed.2014.11.002

[26] Morgan C Martin A Shapiro R. Outcomes after transplantation of deceased-donor kidneys with rising serum creatinine. Am J Transplant. 2007;7:1288–1292. 17359500 10.1111/j.1600-6143.2007.01761.x

