# Supplementary material for: Understanding the Relationship Between Severe Donor Diabetes Insipidus, Kidney Donor Profile Index, and Recipient Kidney Function
Source: Transplant Direct. 2026 Feb 17;12(3):e1916. doi: 10.1097/TXD.0000000000001916 (PMC12915717; doi:10.1097/TXD.0000000000001916)
Supplement: Supplementary file 1 [file txd-12-e1916-s001.pdf]

Table S1. Comparison of KDPI-Peak vs KDPI-Terminal for predicting reduced GFR at 2 years (n=22,221)

	Limited DI (n=15,954)			Severe DI (n=6,267)		
	**OR, HR, coef.**	**95% CI, p-value**	**AIC**	**OR, HR, coef.**	**95% CI, p-value**	**AIC**
**eGFR <60, 2 years**						
KDPI-Terminal			**20673**			**8171**
C	REF			REF		
D	2.32	(2.06, 2.61) <0.001		2.38	(1.94, 2.92) <0.001	
KDPI-Peak			**20659**			**8137**
C	REF			REF		
D	2.13	(1.93, 2.36) <0.001		2.47	(2.07, 2.95) <0.001	
**eGFR <30, 2 years**						
KDPI-Terminal			**9931**			**3483**
C	REF			REF		
D	2.19	(1.90, 2.53) <0.001		2.31	(1.79, 2.98) <0.001	
KDPI-Peak			**9917**			**3480**
C	REF			REF		
D	2.14	(1.88, 2.43) <0.001		2.19	(1.74, 2.76) <0.001	
**eGFR continuous, 2 years**						
KDPI-Terminal			**142522**			**55219**
C	REF			REF		
D	-8.88	(-9.93, -7.83) <0.001		-8.98	(-10.67, -7.29) <0.001	
KDPI-Peak			**142501**			**55184**
C	REF			REF		
D	-8.19	(-9.13, -7.26) <0.001		-9.05	(-10.53, -7.58) <0.001	

Adjusted for: recipient sex, age, BMI, diabetes, PRA, cold ischemia time.
DI: diabetes insipidus; AIC: Akaike Information Criterion; eGFR: estimated glomerular filtration rate; KDPI: kidney donor profile index

Table S2. Effect of KDPI reclassification on graft function at 2 years, KDPI > 35%

	Entire Cohort (n=22,221)			Severe DI (n= 6,267)		
	OR, HR, coef.	95% CI	p-value	OR, HR, coef.	95% CI	p-value
eGFR <60, 2 years	1.77	(1.51, 2.07)	<0.001	2.43	(1.76, 3.36)	<0.001
eGFR <30, 2 years	1.72	(1.39, 2.12)	<0.001	1.69	(1.10, 2.59)	0.016
eGFR continuous, 2 years	-5.98	(-7.43, -4.53)	<0.001	-8.08	(-10.72, -5.44)	<0.001

Models adjusted for: sex, age, BMI, diabetes, PRA, cold ischemia time, KDPI group
DI: diabetes insipidus; eGFR: estimated glomerular filtration rate; KDPI: kidney donor profile index

Table S3. Effect of KDPI Reclassification on Graft Function, KDPI 0-100%

	Entire Cohort (n=57,998)			Severe DI (n= 19,149)		
	**OR, HR, coef.**	**95% CI**	**P-value**	**OR, HR, coef.**	**95% CI**	**P-value**
**EGFR <60, 1 year**	**1.37**	**(1.30, 1.44)**	**<0.001**	**1.41**	**(1.30, 1.53)**	**<0.001**
**EGFR <30, 1 year**	**1.33**	**(1.17, 1.51)**	**<0.001**	**1.30**	**(1.04, 1.62)**	**0.020**
**EGFR, continuous, 1 year**	**-3.50**	**(-4.03, -2.98)**	**<0.001**	**-3.85**	**(-4.74, -2.97)**	**<0.001**
**Graft failure**	1.02	(0.90, 1.15)	0.761	0.81	(0.65, 1.01)	0.056

Models adjusted for: sex, age, BMI, diabetes, PRA, cold ischemia time, KDPI group

DI: diabetes insipidus; eGFR: estimated glomerular filtration rate; KDPI: kidney donor profile index

Table S4. Comparison of KDPI-Peak vs KDPI-Terminal for predicting reduced GFR and graft failure, alternative DI criteria (n=28,718)

	**Limited DI (n=25,288)**			**Severe DI (n=3,430)**		
	**OR, HR, coef.**	**95% CI, p-value**	**AIC**	**OR, HR, coef.**	**95% CI, p-value**	**AIC**
**EGFR <60, 1 year**						
KDPI-Terminal			**32123**			**4521**
C	REF			REF		
D	2.22	(2.02, 2.44) <0.001		2.00	(1.52, 2.63) <0.001	
KDPI-Peak			**32068**			**4510**
C	REF			REF		
D	2.13	(1.97, 2.32) <0.001		2.08	(1.63, 2.66) <0.001	
**EGFR <30, 1 year**						
KDPI-Terminal			**14943**			**1787**
C	REF			REF		
D	2.31	(2.07, 2.59) <0.001		2.45	(1.71, 3.52) <0.001	
KDPI-Peak			**14927**			**1788**
C	REF			REF		
D	2.22	(2.00, 2.46) <0.001		2.23	(1.60, 3.11) <0.001	
**EGFR Continuous, 1 year**						
KDPI-Terminal			**222952**			**30052**
C	REF			REF		
D	-7.98	(-8.76, -7.20) <0.001		-8.39	(-10.73, -6.05) <0.001	
KDPI-Peak			**222891**			**30038**
C	REF			REF		
D	-7.60	(-8.29, -6.91) <0.001		-8.40	(-10.46, -6.33) <0.001	
**Graft failure**						
KDPI-Terminal			**20656**			**1846**
C	REF			REF		
D	1.97	(1.68, 2.32) <0.001		1.23	(0.66, 2.27) 0.516	
KDPI-Peak			**20659**			**1845**
C	REF			REF		
D	1.82	(1.57, 2.11) <0.001		1.36	(0.80, 2.32) 0.260	

Adjusted for: recipient sex, age, BMI, diabetes, PRA, cold ischemia time.
DI: diabetes insipidus; AIC: Akaike Information Criterion; eGFR: estimated glomerular filtration rate; KDPI: kidney donor profile index

Table S5. Effect of KDPI reclassification on graft function, KDPI > 35%, alternative DI criteria

	**Entire Cohort (n=28,718)**			**Severe DI (n= 3,430)**		
	**OR, HR, coef.**	**95% CI**	**p-value**	**OR, HR, coef.**	**95% CI**	**p-value**
**EGFR <60, 1 year**	**1.81**	**(1.58, 2.08)**	**<0.001**	**2.16**	**(1.35, 3.46)**	**0.001**
**EGFR <30, 1 year**	**1.71**	**(1.42, 2.06)**	**<0.001**	1.51	(0.77, 2.98)	0.230
**EGFR, continuous, 1 year**	**-5.79**	**(-6.99, -4.59)**	**<0.001**	**-7.51**	**(-11.42, -3.61)**	**<0.001**
**Graft failure**	**1.37**	**(1.03, 1.81)**	**0.028**	1.66	(0.67, 4.09)	**0.271**

Models adjusted for: sex, age, BMI, diabetes, PRA, cold ischemia time, KDPI group
DI: diabetes insipidus; eGFR: estimated glomerular filtration rate; KDPI: kidney donor profile index
